# Pancreatic Neuroendocrine Tumors in Mice Deficient in Proglucagon-Derived Peptides

**DOI:** 10.1371/journal.pone.0133812

**Published:** 2015-07-20

**Authors:** Yuko Takano, Kenji Kasai, Yoshiko Takagishi, Toyone Kikumori, Tsuneo Imai, Yoshiharu Murata, Yoshitaka Hayashi

**Affiliations:** 1 Department of Genetics, Research Institute of Environmental Medicine, Nagoya University, 464–8601, Nagoya, Aichi, Japan; 2 Department of Transplantation and Endocrine Surgery, Nagoya University Graduate School of Medicine, 466–8550, Nagoya, Aichi, Japan; 3 Department of Pathology, Aichi Medical University, 480–1195, Nagakute, Aichi, Japan; 4 Department of Breast and Endocrine Surgery, Aichi Medical University, 480–1195, Nagakute, Aichi, Japan; La Jolla Institute for Allergy and Immunology, UNITED STATES

## Abstract

Animal models with defective glucagon action show hyperplasia of islet α-cells, however, the regulatory mechanisms underlying the proliferation of islet endocrine cells remain largely to be elucidated. The *Gcg^gfp/gfp^* mice, which are homozygous for glucagon/green fluorescent protein knock-in allele (GCGKO), lack all proglucagon-derived peptides including glucagon and GLP-1. The present study was aimed to characterize pancreatic neuroendocrine tumors (panNETs), which develop in the GCGKO mice. At 15 months of age, macroscopic GFP-positive tumors were identified in the pancreas of all the GCGKO mice, but not in that of the control heterozygous mice. The tumor manifested several features that were consistent with pancreatic neuroendocrine tumors (panNETs), such as organoid structures with trabecular and cribriform patterns, and the expression of chromogranin A and synaptophysin. Dissemination of GFP-positive cells was observed in the liver and lungs in 100% and 95%, respectively, of 15-month-old GCGKO mice. To elucidate the regulatory mechanism for tumor growth, PanNET grafts were transplanted into subrenal capsules in GCGKO and control mice. Ki-67 positive cells were identified in panNET grafts transplanted to GCGKO mice 1 month after transplantation, but not in those to control mice. These results suggest that humoral factors or conditions specific to GCGKO mice, are involved in the proliferation of panNETs. Taken together, GCGKO mice are novel animal model for studying the development, pathogenesis, and metastasis panNETs.

## Introduction

Glucagon is produced in islet α-cells through cleavage of proglucagon by prohormone convertase 2 (Pcsk2) and increases glucose production in liver, the major target organ of glucagon [1–3]. Aberrant α-cell proliferation and dysregulated glucagon secretion are postulated to play important roles in the pathogenesis of diabetes mellitus [4], however, the regulatory mechanisms that control islet α-cell mass are not well understood [5, 6]. Studies using animal models have demonstrated that deficiency of glucagon induces proliferation of α-cells. Mice deficient in glucagon receptors (*Gcgr* ^*-/-*^), or prohormone convertase 2 (*Pcsk2* ^*-/-*^) develop hyperplasia of α-cells [7, 8]. Both *Gcgr* ^*-/-*^ and *Pcsk2* ^*-/-*^ mice show lower blood glucose levels and increased serum GLP-1 levels than control mice. It is not clear whether changes in glucose and/or GLP-1 levels are prerequisites for α-cell proliferation [5, 6]. On the other hand, the mice with liver-specific ablation of *Gcgr* or stimulatory α subunits of G-protein also develop hyperplasia of α-cells, indicating that glucagon does not suppress α cell proliferation by itself but does so as a consequence of its action in the liver [9–11].

Recently, we established a mouse model in which the glucagon gene is disrupted by the insertion of GFP. In heterozygous mice (*Gcg* ^*gfp/+*^), proglucagon-derived peptides and GFP are expressed in pancreatic α-cells and intestinal L-cells. Homozygous mice (*Gcg* ^*gfp/gfp*^), hereafter referred to as GCGKO mice, lack all proglucagon-derived peptides, including glucagon and GLP-1. GCGKO mice show hyperplasia of GFP-positive α-like cells, which contain GFP but not glucagon [12]. In contrast to preceding animal models such as *Gcgr* ^*-/-*^ and *Pcsk2* ^*-/-*^ mice, adult GCGKO mice are normoglycemic and lack GLP-1. Therefore, in GCGKO mice, neither GLP-1 nor sustained low blood glucose levels are required for the development of hyperplasia of α-like cells [6].

It has been reported that *Gcgr* ^*-/-*^ mice develop multiple pancreatic neuroendocrine tumors (panNETs) and, in particular, glucagonomas [13, 14]. PanNETs account for approximately 1–2% of clinically detected pancreatic neoplasms in humans, and most human panNETs are sporadic well-differentiated tumors. Multiple tumors are rare, except for those identified in patients with the multiple endocrine neoplasms type 1 (MEN1) or von Hippel-Lindau (VHL) syndromes [15, 16]. Hyperplasia of α-cells has also been reported in humans [17, 18] and a patient harboring a homozygous GCGR mutation (P86S) has been identified, who showed hyperglucagonemia without the glucagonoma symptoms, diffuse α-cell hyperplasia, and pancreatic neuroendocrine tumors [19].

To address regulatory mechanism for panNETs development, we characterized the progression of hyperplasia of α-like cells and the development of panNETs in GCGKO mice in the present study. We analyzed the regulatory mechanisms of panNETs cell proliferation by a subrenal capsule assay for panNET allografts.

## Materials and Methods

### Animals

The establishment of the GCGKO mouse model has been previously described [12]. All mice were maintained in specific pathogen-free barrier facilities at the Research Institute of Environmental Medicine, Nagoya University, under a constant controlled temperature and a 12/12-h dark/light cycle. Food (standard chow) and water were available *ad libitum*. This study was carried out in strict accordance with the recommendations in the Guide for the Care and Use of Laboratory Animals of the National Institutes of Health. The protocol was approved by the Nagoya University Institutional Animal Care and Use Committee (Permit Number: 12061, 12187, 13061, 13187 and 14025). All surgery was performed under sodium pentobarbital anesthesia, and all efforts were made to minimize suffering. All experiments were performed using male mice. Wild-type *Gcg* ^*+/+*^ and heterozygous *Gcg* ^*gfp/+*^ mice served as the controls. Animals were inspected daily for their home cage activity and response of animals to tail suspension was examined twice a week by researchers adequately experienced or trained in the recognition of weakness in the response. Body weight was measured weekly. Animals that showed either body weight decrease greater than 10% of their peak body weight or weakness in response to tail suspension were inspected daily thereafter for their body weight and righting reflex. Criteria for euthanasia as humane endpoints were based on body weight decrease greater than 2 g within 24 hours and/or failure to right within 3 seconds when animals were set in lateral position. Animals were euthanized by CO_2_ asphyxiation. Every animal euthanized or found unexpectedly dead was necropsied and were represented as deaths to estimate survival rate.

### Morphology, histology, antibodies, and immunohistochemistry

Images of the mice and their organs were obtained using a digital fluorescent microscope (VB-6000; Keyence Japan, Osaka, Japan, or Leica AF6500; Leica Microsystems, Tokyo, Japan). For histological analyses, tissues were fixed in 10% formalin solution and embedded in paraffin for hematoxylin and eosin (H&E) staining. Alternatively, tissues were fixed by perfusion with 4% paraformaldehyde. Frozen sections were the cut using a Microm HM500 OM Microtome Cryostat (Carl Zeiss Japan, Tokyo, Japan).

The following primary antibodies were used for immunohistochemical analysis: guinea pig anti-insulin, rabbit anti-glucagon, rabbit anti-somatostatin, goat anti-pancreatic polypeptide, rabbit anti-chromograninA (cgA), rabbit anti-Ki67, and rabbit anti-VEGF (Abcam, Tokyo, Japan). Alexa568- or Cy3- labeled species-specific anti-IgG antibodies (Life Technologies Japan, Tokyo, Japan) were used as secondary antibodies. Images were obtained using a LSM710 laser scanning microscope (Carl Zeiss Japan), or a HS BZ-9000 fluorescent microscope system (Keyence). The Ki-67 index was calculated by dividing the total number of nuclei by the number of Ki-67-positive nuclei. The number of nuclei was counted by observing 8–10 fields with a 40× lens.

### Isolation of islets

Pancreatic islets were isolated from 2–4-month-old mice using the collagenase digestion method, followed by hand-picking [20]. Seventy to 120 islets were isolated from each pancreas.

### RNA extraction and analyses of gene expression

Total RNA was isolated from the islets of GCGKO and *Gcg* ^*gfp/+*^ mice, pancreatic tumors, and metastatic liver tumors, using an RNeasy Mini Kit (Qiagen, Tokyo, Japan), according to the manufacturer’s instructions. cDNAs were synthesized and quantitative real-time RT-PCR was performed using Rotor-Gene Q (Quiagen) with SyBr Green, or using the ABI StepOne Real-Time PCR System (Life Technologies Japan) and Taqman Gene Expression Assays. The detailed procedure has been described previously [21] and the specific primer sequences are available on request.

### Tumor graft transplantation by subrenal capsule assay (SRCA)

After excision of the pancreatic tumors from 15-month-old GCGKO mice, the tumors were minced using scissors (to yield grafts of about 1mm^3^). Approximately 2 μg of these grafts were then transplanted into the left subrenal capsule of 2-month-old GCGKO mice or *Gcg* ^*gfp/+*^ mice. Four weeks later, the mice were sacrificed for histological examination. The Ki-67 labeling index was calculated as explained above by observing 6–8 fields with a 40× lens.

### Statistical analysis

Data are presented as the mean ± SEM. Significance was evaluated using Student's t-test or ANOVA. Differences were considered statistically significant when P-values were < 0.05.

## Results

### Hyperplasia of α-like cells and the development of pancreatic tumors in GCGKO mice

**Fig 1:**
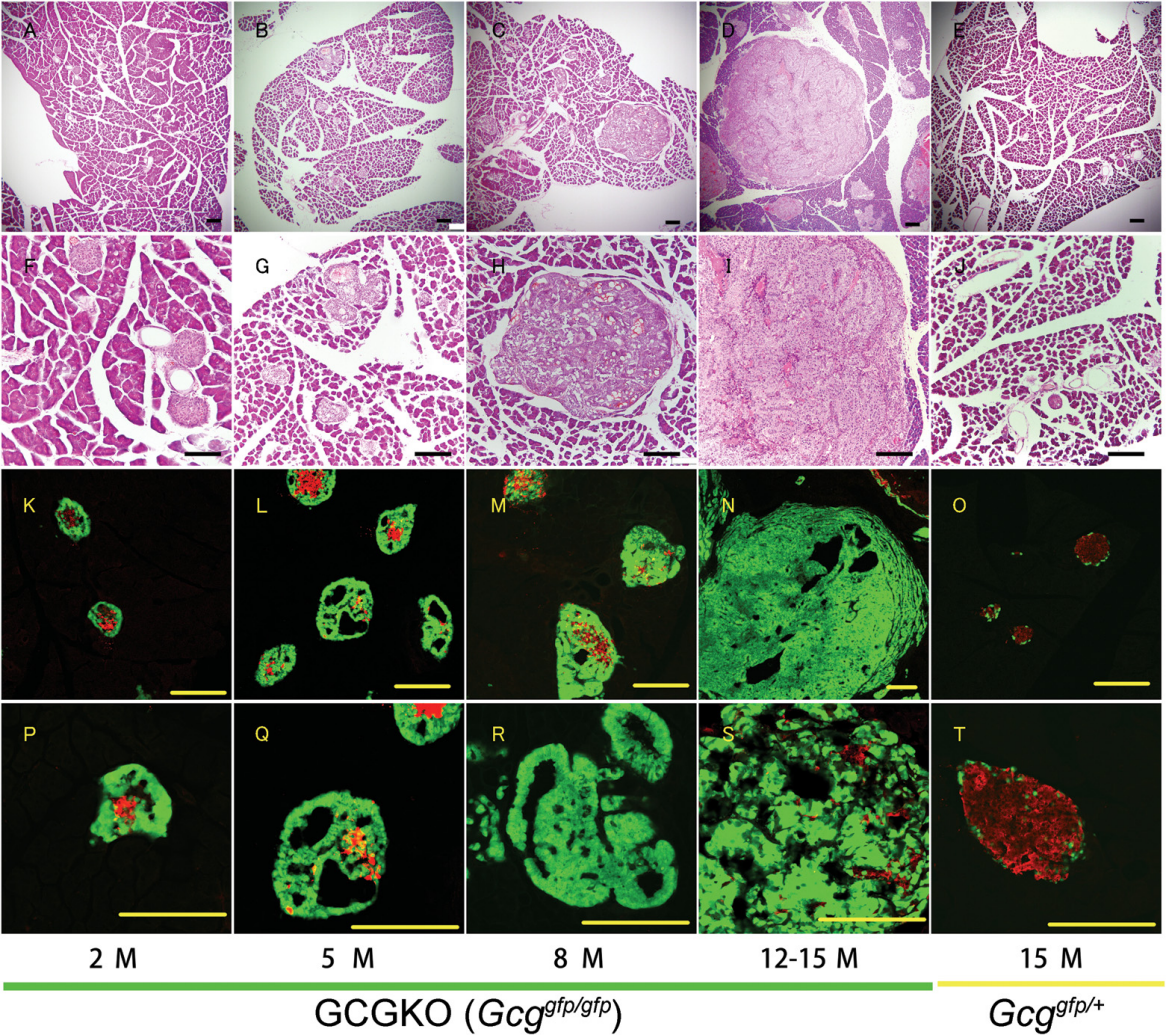
Hyperplasia of α-like cells and neuroendocrine tumors in the GCGKO mouse model. H&E-staining (A–J) and fluorescent immunohistochemical analyses (K–T) of the pancreas. Sections of pancreas from 2-month-old (A, F, K, and P), 5-month-old (B, G, L, and Q), 8-month-old (C, H, M, and R) and 12–15-month-old (D, I, N, and S) GCGKO mice and from 15-month-old Gcg^gfp/+^ mice (E, J, O, and T) are shown. Immunoreactivity for insulin is red, and the fluorescent signal generated by GFP is green. Scale bars: 200 μm.

**Fig 2:**
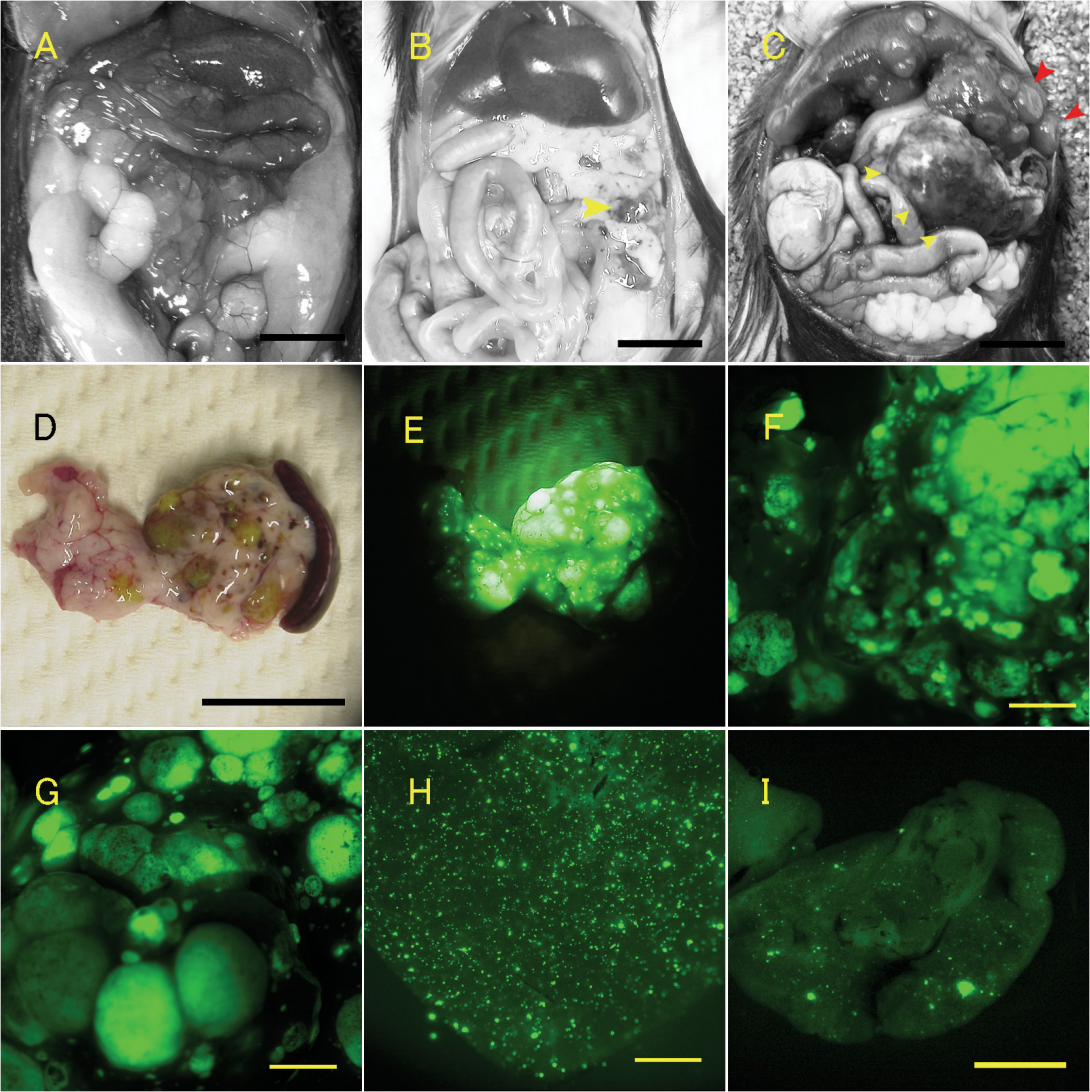
Macroscopic images of the tumor and disseminated tumor cells. A–C: Macroscopic images of abdominal organs from an 18-month-old control mouse (A) and a 15-month-old GCGKO (B and C) mouse. Pancreatic tumors are indicated by yellow arrow heads and macroscopic liver tumors by red arrow heads. D and E: Images of dissected pancreas and spleen. Brightfield (D) and fluorescent (E) images are shown. Scale bars: 10 mm (in A–D). F–I: Fluorescent images of the tumor and disseminated GFP-positive cells. Macroscopic tumors in the pancreas (F) and liver (G), and microscopic dissemination of GFP-positive cells in the liver (H) and lungs (I). Scale bars: 2.5 mm (in F–I).

In the islets of *Gcg* ^*gfp/+*^ mice, the GFP-positive α-cells formed a monolayered mantle surrounding insulin-positive ß-cells. By contrast, at 2 months of age, the GCGKO mice showed an increased number of GFP-positive cells, which still formed a thickened and multilayered mantle surrounding the insulin-positive ß-cells, indicating hyperplasia of α-like cells (Fig 1A, 1F, 1K and 1P). At 5 months of age, the number of α-like cells increased further, and trabecular structures developed (Fig 1B, 1G, 1L and 1Q). At 8 months of age, the size of the islets increased, and some had formed a tumor-like mass (1–2 mm in diameter; Fig 1C, 1H, 1M and 1R). At 12 months of age, the size of the islets increased further (2–5 mm in diameter; Fig 1D, 1I, 1N and 1S), and GFP-positive cells were detected in the liver by fluorescent microscopy, suggesting micro-metastasis (see Fig 2H). The survival rates of the GCGKO and control mice at the age are 66% and 99%, respectively. At 15 months of age, no hyperplasia or tumor-like islet masses were detected in the *Gcg* ^*gfp/+*^ mice (Fig 1E, 1J, 1O and 1T) or wild-type mice (data not shown).

Table 1 summarizes the macroscopic characteristics of the tumors in mice at 15 months of age. The survival rates of the GCGKO and control mice at the age are 44% and 99%, respectively. Multiple GFP-positive pancreatic tumors were detected in GCGKO mice (Fig 2B and 2D–2F), but not in *Gcg* ^*gfp/+*^ or wild-type mice (Fig 2A). GFP-positive metastatic tumors of either macroscopic or microscopic size (Fig 2C, red allow heads) were identified in the liver (42% or 100%, respectively) and lungs (5.2% or 95%, respectively) of the GCGKO mice (Table 1 and Fig 2G–2I). No GFP-positive cells or tumors were detected in other organs such as the brain, thyroid, kidney, and adrenal glands, with the exception of endocrine cells in the gastrointestinal tracts (data not shown).

**Table 1:** Macroscopic characteristics of the tumors.

	GCGKO (*Gcg* ^*gfp/gfp*^)	*Gcg* ^*gfp/+*^	Wild type	P- Value
Numbers observed	19	18	11	
Age (month)	15.5±0.9	15.6±1.3	15.5±1.0	0.528
Body weight (g)	37.9±3.5	39.1±5.2	40.8±6.8	0.320
With pancreatic tumor	19/19 (100%)	0/18 (0%)	0/11 (0%)	<0.001
Size of pancreatic tumor (mm)	14.2±5.4 (8–20)	—	—	
Macroscopic liver tumors	8/19 (42%)	0/18 (0%)	0/11(0%)	0.001
Macroscopic lung tumors	1/19 (5.2%)	0/18 (0%)	0/11(0%)	0.459
Microscopic GFP-positive foci in liver	19/19 (100%)	0/18 (0%)	0/11(0%)	<0.001
Microscopic GFP-positive foci in lung	18/19 (95%)	0/18 (0%)	0/11(0%)	<0.001

### Characterization of panNETs in GCGKO mice

**Fig 3:**
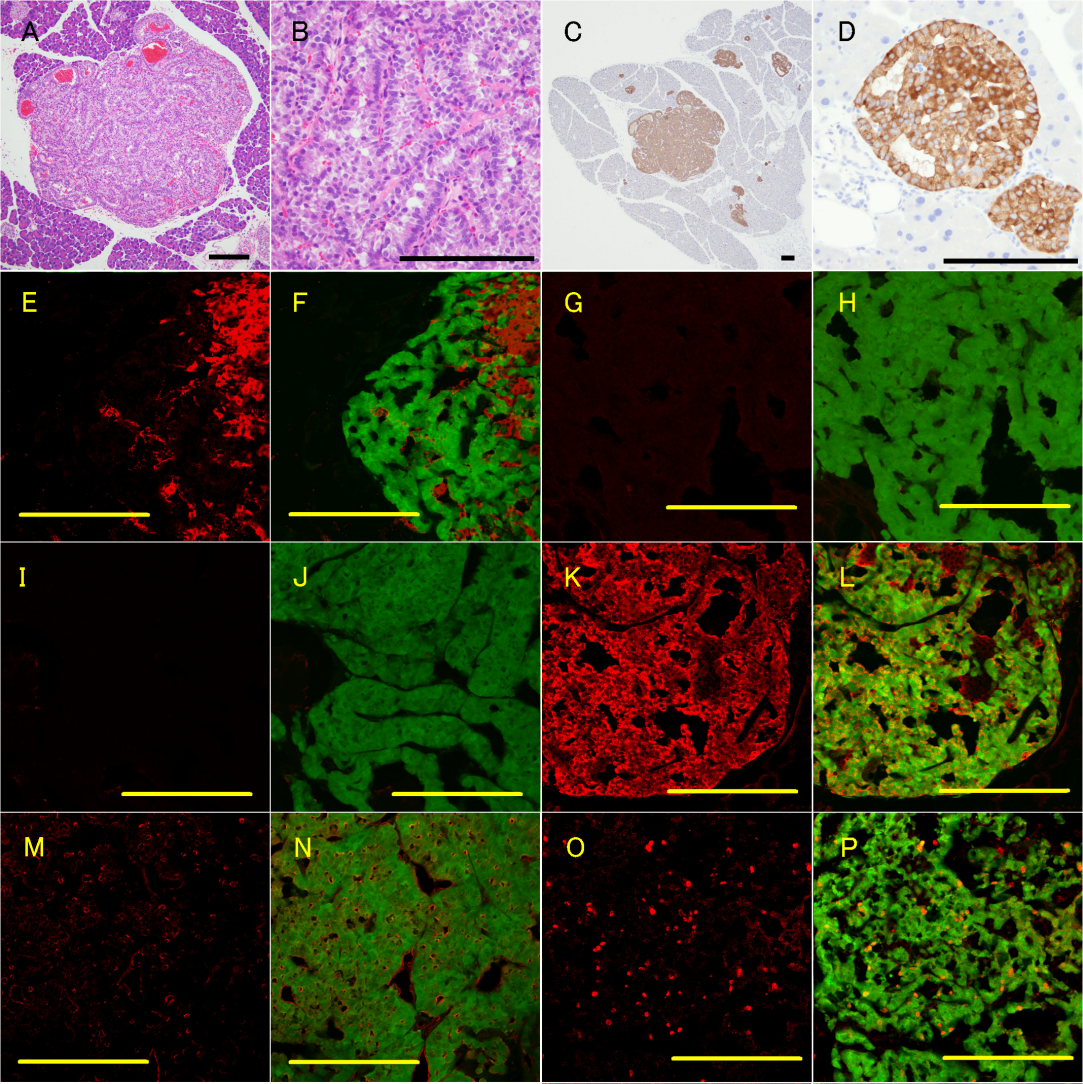
Neuroendocrine tumors in the pancreas of 15-month-old GCGKO mice. A–D: H&E-staining (A and B) and immunohistochemical analysis of synaptophysin expression (C and D) in the tumor. E–P: Fluorescent immunohistochemical analyses showing the GFP fluorescent signal (F, H, J, L, N, and P) or not (E, G, I, K, M, and O). Immunoreactivity for insulin (E and F), somatostatin (G and H), pancreatic polypeptide (I and J), chromogranin A (K and L), VEGF (M and N), and Ki-67 (O and P) is shown in red. Scale bars: 200 μm.

The pancreatic tumors showed a circumscribed border with no fibrotic capsule. Numerous small vessels were detected in the tumors, and areas of hemorrhage were occasionally seen. The tumor cells formed an organoid structure, including nesting, trabecular, and cribriform patterns, which are typical features of panPET. The tumor cells were polygonal, with an eosinophilic or amphophilic cytoplasm, and nuclei located at the periphery (Fig 3A and 3B). Immunohistochemically, the tumor cells stained positive for synaptophysin (Fig 3C and 3D) and chromogranin A (Fig 3K and 3L), which is consistent with human panPETs. Insulin-positive cells were associated with the tumor, and cells positive for both GFP and insulin were also present at very low levels (Fig 3E and 3F, arrows). No cells stained positive for somatostatin (Fig 3G and 3H) or pancreatic polypeptide (Fig 3I and 3J). Immunoreactivity of vascular endothelial growth factor (Fig 3M and 3N) were detected along the trabecular structures, as well as the sinusoidal structure in the tumor, indicating that the panNET was hypervascularized. The labeling index of the cell proliferation marker, Ki-67, was 4.5% (range 3.4–6.8%; Fig 3O and 3P, and data not shown).

### Characterization of metastatic tumors in the liver

**Fig 4:**
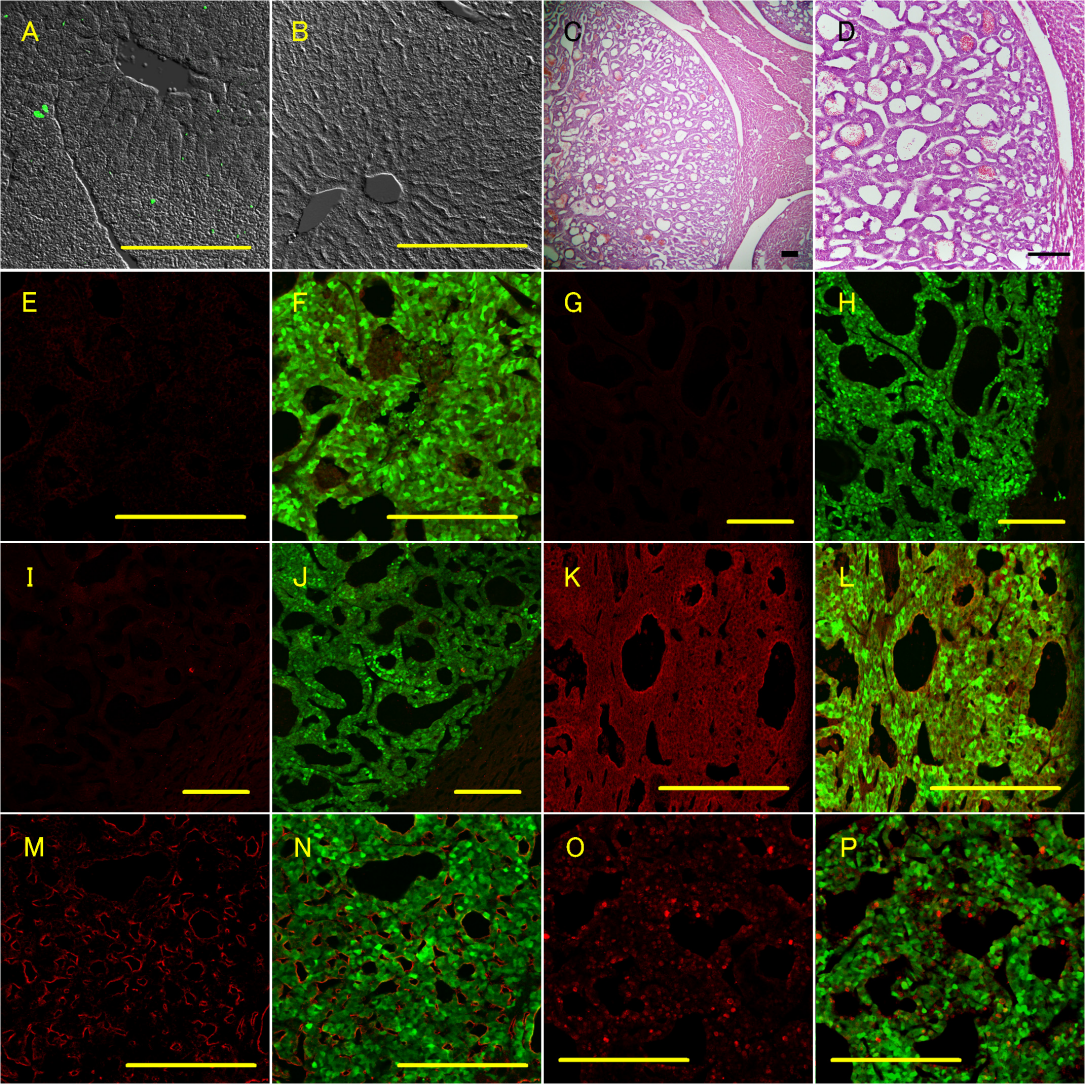
Dissemination of GFP-positive cells and metastatic neuroendocrine tumors in the liver of GCGKO mice. A and B: Fluorescence with phase contrast imaging of the liver section. Livers from a 12-month-old GCGKO mouse (A) and an 18-month-old Gcg^gfp/+^ mouse (B) are shown. C and D: H&E-staining of the metastatic neuroendocrine tumor. E–P: Fluorescent immunohistochemical analyses showing the GFP fluorescent signal (F, H, J, L, N, and P) or not (E, G, I, K, M, and O). Immunoreactivity for insulin (E and F), somatostatin (G and H), pancreatic polypeptide (I and J), chromogranin A (K and L), VEGF (M and N), and Ki-67 (O and P) is shown in red. Scale bars: 200 μm.

Dissemination of GFP-positive cells in the liver was identified in some of the 12-month-old GCGKO mice and in all the 15-month-old GCGKO mice (Fig 4A and Table 1). GFP-positive cells were absent from the livers of the *Gcg* ^*gfp/+*^ mice (Fig 4B and Table 1). The macroscopic GFP-positive tumors in the liver were well-circumscribed. Microscopically, these metastatic tumor cells showed an organoid structure, as seen in the pancreata, and were separated from the hepatic parenchyma by fibrous capsules (Fig 4C and 4D). No cells in the metastatic tumors stained positive for insulin (Fig 4E and 4F), somatostatin (Fig 4G and 4H), or pancreatic polypeptide Y (Fig 4I and 4J). Expression patterns of chromogranin A (Fig 4K and 4L) and vascular endothelial growth factor (Fig 4M and 4N) in the metastatic tumors were similar to those observed in pancreatic tumors. Ki67-positive cells were also identified in the liver (Fig 4O and 4P), although the labeling index appeared to be lower than that of the pancreatic tumor (Fig 3O and 3P).

### Gene expression in the panNETs

**Fig 5:**
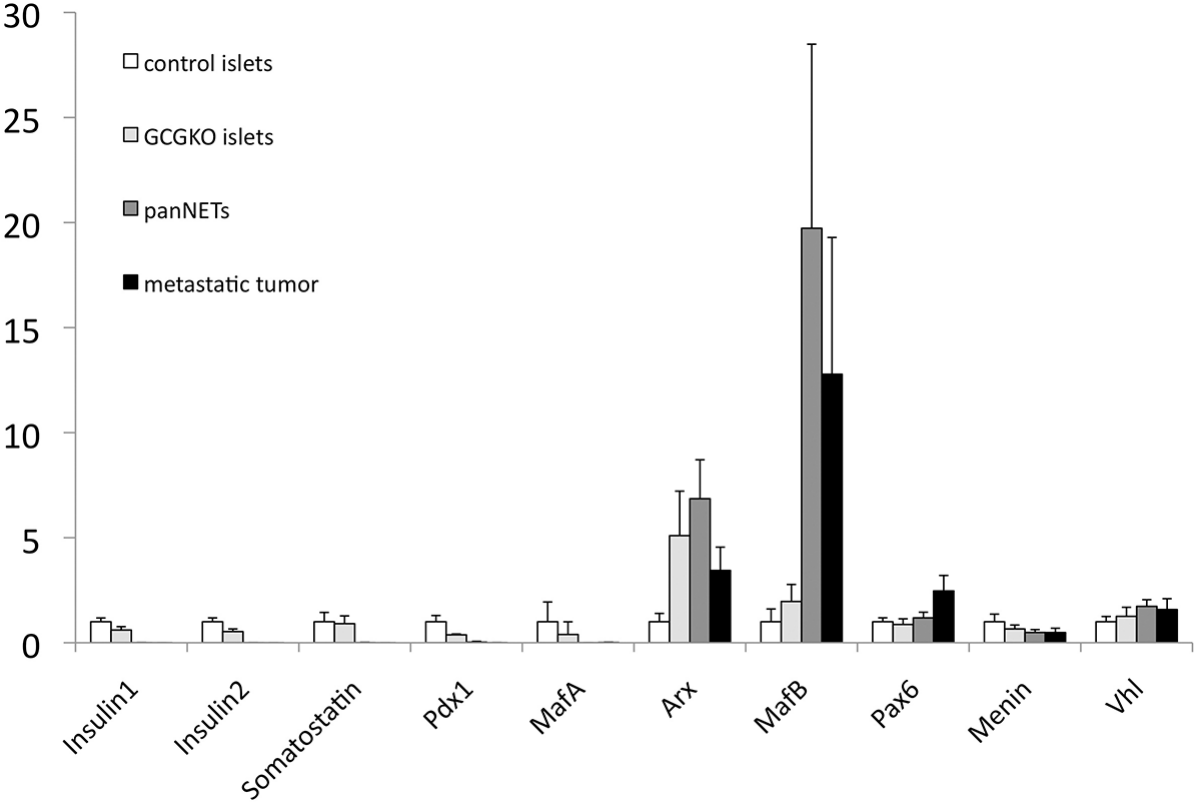
Gene expression in the islets and tumors. Relative expression levels of the indicated genes normalized to ß-actin are shown. Expression levels in control islets are set as 1. Data represent the mean±SD. N = 4–8 (islets: 6, panNET: 8, and metastatic tumor in liver: 4).

To further characterize the panNETs, we analyzed gene expression in the panNETs and the metastatic tumors in the liver together with islets isolated from the control and GCGKO mice (Fig 5). The genes encoding insulin and somatostatin were expressed at very low levels in the panNETs. mRNA for Pdx-1 and MafA, transcription factors that are exclusively expressed in the ß-cells of adult mice [22, 23], was detected at very low levels in the panNETs. In addition, three transcription factors that are expressed in α-cells, Arx, MafB, and Pax6 [22, 24], were detected in the panNETs, which indicates that the panNETs maintain α-cell-like characteristics. Two anti-oncogenes, Vhl and Menin, were expressed in the panNETs at a level comparable with that in the control and GCGKO islets.

### Tumor graft transplantation in the subrenal capsule

**Fig 6:**
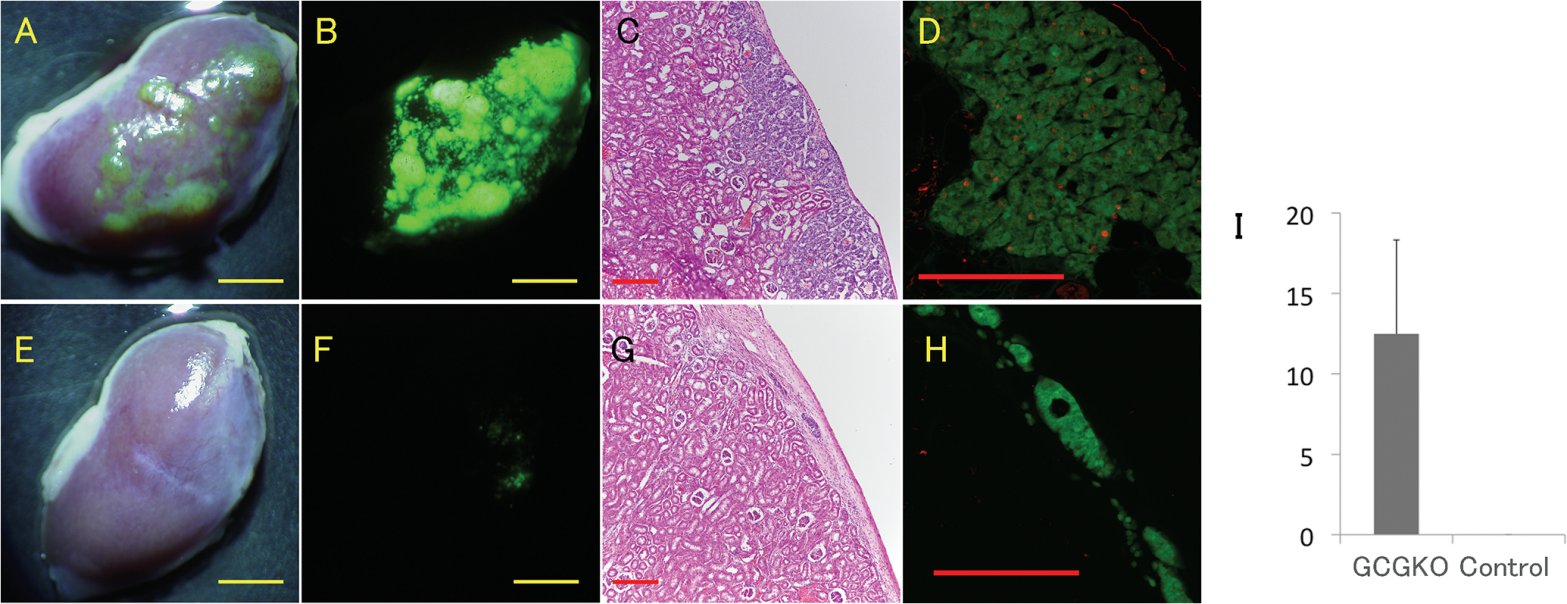
Subrenal capsule transplantation of the pancreatic neuroendocrine tumors. Tumor grafts were transplanted into GCGKO (A–D) and control Gcg^gfp/+^ mice (E–H) and analyzed 4 weeks after transplantation. Macroscopic (A and E) and fluorescent (B and F) images of the kidney are shown. Scale bars: 2.5 mm. H&E-staining (C and G) and fluorescent immunohistochemistry for Ki67 (D and H) are shown. Ki-67 immunoreactivity is red, and GFP fluorescence is green. Scale bars: 200 μm. (I) Ki-67 index of the transplants in GCGKO and control mice. Data represent the mean±SD. N = 6 (GCGKO) and 4 (control).

To address whether the growth of the tumor depends on the presence or absence of specific humoral factors in GCGKO mice, panNETs were transplanted in to the subrenal capsule of GCGKO or control *Gcg* ^*gfp/+*^ mice. Fig 6A and 6B show tumor grafts transplanted into the GCGKO mice, which formed macroscopic GFP-positive tumors. The tumors showed an organoid structure (Fig 6C), and Ki-67-positive cells were frequently identified (Fig 6D). The mass of the tumor grafts did not increase in the control mice (Fig 6E–6G), and very few, if any, Ki-67-positive cells were identified (Fig 6H). The Ki-67 labeling index of the tumors that were transplanted into the GCGKO mice was significantly higher (12±5.8%, n = 6, p = 0.013), compared with 0±0% (n = 4) for the control mice (Fig 6I). These results indicate that growth of the tumor graft in the subrenal capsule was dependent on the humoral conditions specific to the GCGKO, such as the presence of growth-promoting factors, and the absence of growth inhibitory factors, or both.

## Discussion

The present study showed that panNETs in GCGKO mice arose from hyperplasic α-like cells, and that the tumor cells frequently disseminated and formed metastatic foci in the liver and lungs. Hyperplasia of α-cells and the development of panNETs have been reported in *Gcgr* ^*-/-*^ mice and in humans harboring glucagon receptor mutations. However, tumor metastasis was not so frequently observed in animal models and never in human cases. Therefore, the GCGKO mouse serves as a novel animal model in which to study the dissemination and metastasis of panNETs.

*Gcgr* ^*-/-*^ mice show increased GLP-1 levels and lower blood glucose levels than control mice, whereas GCGKO mice lack GLP-1 and are normoglycemic [6, 7, 12]. Therefore, one of the possible reasons for the difference in the character of panNETs between GCGKO and *Gcgr* ^*-/-*^ mice is the absence or presence of GLP-1.

GLP-1 promotes ß-cell proliferation, stimulates insulin secretion, and suppresses glucagon secretion from α-cells [25]. It is controversial whether GLP-1 receptor is expressed in α-cells, and it is unclear whether GLP-1 directly regulates α-cell proliferation. The proliferation of α-cell-derived αTC1 cells is stimulated by RNAi-mediated Gsα ablation and suppressed by forskolin [26]. Therefore, it is possible that GLP-1 increases intracellular cAMP in α-cells and suppresses its proliferation. If this is the case, absence of GLP-1 from GCGKO mice may have aggravated aberrant proliferation of α-like cells. Double knock-out mice for the Gcgr and GLP1 receptors (*Gcgr* ^*-/-*^ *Glp1r* ^*-/-*^) are normoglycemic, lack most (if not all) GLP-1 activity, and develop α-cells hyperplasia [27]. It is not known whether *Gcgr* ^*-/-*^ *Glp1r* ^*-/-*^ mice develop panNETs. Comparative studies of panNETs in *Gcgr* ^*-/-*^ and *Gcgr* ^*-/-*^ *Glp1r* ^*-/-*^ mice should clarify whether GLP-1 suppresses the growth of panNETs.

Accumulating data suggest that the metabolic status of the liver plays a key role in regulating the proliferation of islet endocrine cells. Liver-specific ablation of insulin receptor or glucagon receptors induces the proliferation of islet ß- or α- cells, respectively [9, 28]. Parabiosis or islet transplantation experiments were carried out in these studies, and the results suggested the involvement of humoral signals in regulating the proliferation of islet endocrine cells [28]. On the other hand, it has been reported that neuronal signals evoked by activation of Erk kinase in liver also regulate the proliferation of ß-cells [29]. In the present study, data from the transplantation experiments suggested that humoral conditions specific to GCGKO mice play an important role in the proliferation of transplanted panNET cells.

Administration of insulin antagonists induces the expression of betatrophin in the liver and the proliferation of ß-cells [30]. In addition, forced expression of betatrophin, also referred to as ANGPTL8/Lipasin/RIFL [31–33], induces the proliferation of ß-cells [30]. The panNET cells in the GCGKO model should be useful for screening “alpha-trophin” and/or “alpha-statin”, which regulate the growth of α/α-like cells. Identifying such factors should contribute to the development of methods to suppress both panNET growth and α-cell proliferation in patients with diabetes mellitus.
